# Severe ischemic complications in Covid-19—A case series

**DOI:** 10.1016/j.ijscr.2020.09.009

**Published:** 2020-09-10

**Authors:** Eisa Lari, Ali Lari, Shamlan AlQinai, Mahdi Abdulrasoul, Salman AlSafran, Ahmad Ameer, Salman Al-Sabah

**Affiliations:** General Surgery Department, Jaber Al-Ahmad Hospital, Ministry of Health, Kuwait

**Keywords:** Acute limb ischaemia, Covid-19, Thrombosis, Revascularisation

## Abstract

**Introduction:**

Covid-19 has the propensity to result in a wide array of manifestations. Recently, thromboembolic complications of Covid-19 have been denoted in literature. We report 5 cases of Covid-19 positive patients with no significant comorbidities who developed 1^st^ time episodes of either; acute limb ischemia or bowel ischemia.

**Material and methods:**

This is a retrospective observational study analyzing the clinical characteristics and outcomes of five Covid-19 positive patients. Five patients aged 38–60 presented to our institution from 1^st^ April to 1^st^ June and were diagnosed with Covid-19 pneumonia, subsequently developing severe vascular complications. None of our patients included had any history of thromboembolism nor risk factors that could justify the presentations.

**Presentation of cases:**

Patient 1; presented with bowel ischemia as a first presentation. Patient 2 and 3 developed unsalvageable lower limb ischemia secondary to partial to complete occlusion of infra-renal aorta. Patient 4 presented with acute upper limb ischemia due to complete occlusion of the axillary and brachial artery. Patient 5 developed bilateral lower limb unsalvageable ischemia secondary to aortic bifurcation occlusion. All patients tested Covid-19 positive upon admission, and received standard care.

**Discussion and conclusion:**

The thromboembolic complications seen in our cases were devastating and resulted in significant mortality and morbidity. All vessels affected were medium-large vessels. None of our cases had any significant predisposing medical conditions or history of thromboembolic or ischemic events. A high index of suspicion is necessary when evaluating such patients regardless of thromboembolic history. Appropriate anticoagulation regimens are essential. Our cases add to the currently increasing severe thromboembolic complications of Covid-19.

## Highlights

• Covid-19 has a propensity to cause thromboembolic complications.
• Aggressive anticoagulation has been recommended.
• Despite anticoagulation, some patients may still develop significant thromboembolic complications.
• Medium to large vessel disease resulting in limb ischemia.
• Covid-19 may cause thrombosis in patients with no apparent history or risk factors for thromboembolic disease.

## 1 Introduction

Covid-19 seems to have the propensity to result in a wide array of manifestations. It was declared a pandemic by the World Health Organization in March 2020 [5]. In recent observational studies, an association between venous and arterial thromboembolic disease and inflammatory response caused by Covid-19 has been linked [1,2]. Yet the exact pathophysiology, incidence and prevalence have not been precisely described.

Potentially lethal Covid-19 associated hypercoagulable states have been described [9,10]. Klok et al. describes a thrombotic complication incidence of 31%; primarily venous, in ICU patients despite anticoagulation prophylaxis [3]. Lodigiani et al., described a remarkably increased incidence of arterial and venous vessel disease associated with increased mortality rates [4]. Anticoagulation with heparin has been recommended and associated with decreased mortality rates [[7], [8], [9],^11^]. Similarly to the study we propose, evidence of medium to large vessel thrombotic complications have been reported in patients as young as 33 years old [6].

The aim of our study is to describe the characteristics, course and outcomes of patients who are Covid-19 positive and presenting with unexpected or unexplained arterial and venous vessel disease. This research has been reported in line with the PROCESS criteria [12]. This study was registered as a case series on the www.researchregistry.com website with UIN 5853.

## 2 Methods and material

We report 5 cases of ischemic vessel disease in patients aged 36–60 to our healthcare services in Kuwait from the 1^st^ April 2020 to 1^st^ June 2020. All cases were diagnosed with Covid-19 pneumonia and tested positive via polymerase chain reaction (PCR). It is important to note that all patients were tested for Covid-19 as per strict hospital policies.

All of our patients received airway and breathing support along with recommended regimens of anticoagulation. Furthermore all patients were tested for coagulopathies by serological testing and were negative with low/clinically insignificant titers. All patients received anticoagulation and were followed with coagulation markers; international normalized ratio (INR) and activated partial thrombosplastin time (APTT).

We included patients who did not have a history of coagulopathy or significant comorbidities that can justify such presentations. None of the patients had any history of previous thromboembolism, malignancy, long-haul travel or prolonged immobilization. Patients who had peripheral distal extremity ischemia were not included. Any patient with a prior history of thromboembolic or was at high risk of thromboembolic disease was not included.

## 3 Results

### 3.1 Case 1

A 38 year old male with no identifiable co-morbidities presented to the emergency department complaining of a 2-day history of progressively worsening abdominal pain, nausea, intractable vomiting and shortness of breath. Examination reveals tachycardia, respiratory distress, and abdominal pain out of proportion to the palpation. Apart from mild leukocytosis and a lactate of 2.2 mmol/L, D-Dimer 2100 ng/ml. All other laboratory investigations were normal. After normal plain films of the abdomen and chest, the patient underwent a computed tomography (CT) scan of the abdomen with contrast.

The scan revealed extensive thrombosis of the portal, splenic, superior and inferior mesenteric veins. The mid-portion of the small bowel was suggestive of venous ischemia. No pneumatosis intestinalis was noted. An incidental finding of a totally occluded right pulmonary artery consistent with a pulmonary embolism was noted.

**Fig. 1:**
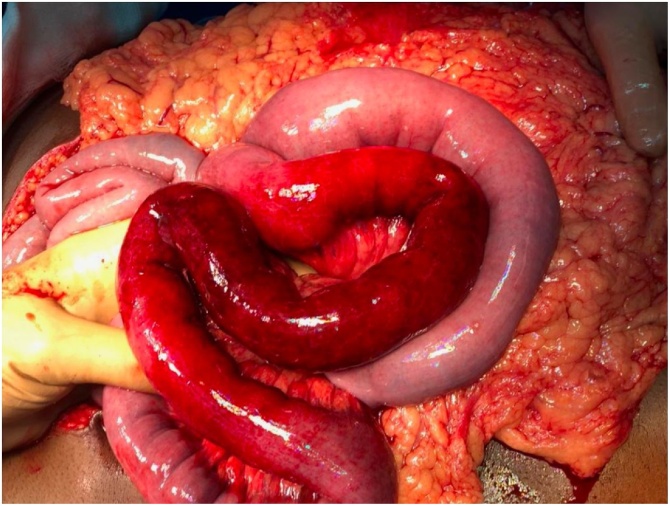
Intraoperative photograph of markedly dusky and inflamed bowel.

The patient received heparin therapy prior to a diagnostic laparoscopy. Intra-operatively a markedly dusky jejunal segment was identified along with turbid fluid in all quadrants. Conversion to open laparotomy ensued followed by resection of the diseased segment with temporary abdominal closure (Fig. 1). The patient was admitted to the ICU for resuscitation and anticoagulation. The patient was later taken to theatre for a side to side anastomosis. The patient was subsequently placed on extracorporeal membrane oxygenation (ECMO) and is currently under care in the intensive care unit (ICU).

### 3.2 Case 2

A 48 year old male with a history of asthma presented to our institution with a cough, shortness of breath and a fever. The patient subsequently tested positive for Covid-19 and was intubated and admitted to the ICU with hypoxic respiratory failure after failure to improve with initial therapy. The patient was started on therapeutic enoxaparin treatment.

**Fig. 2:**
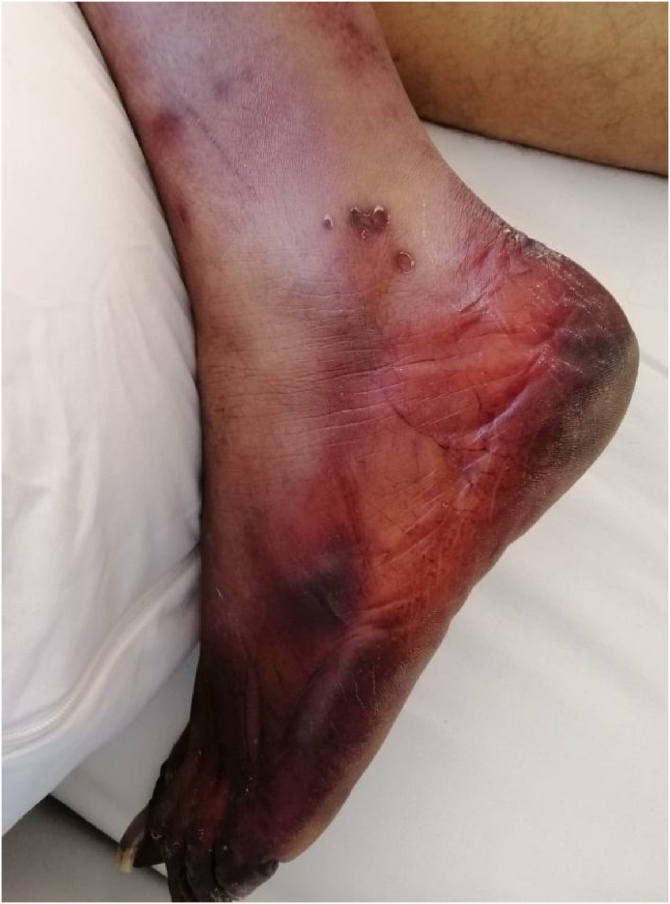
Ischemic left foot, with patchy areas of gangrene.

On the 10th day the patient was on high ventilator settings and minimal inotropic support. On examination, lower limbs showed unsalvageable ischemia with no palpable pulses (Fig. 2). Urgent CT angiography revealed complete occlusion of the left arterial tree from the aortic bifurcation descending down to the infra-popliteal arteries. Contralaterally, distal occlusion of the superficial femoral artery down to the infra-popliteal arteries. CT of the lung displayed ground glass appearance of the lungs associated with consolidation. After consulting the next of kin and the risk; benefit of proceeding to surgery. The joint decision to provide supportive care was made. The patient was declared dead on the same day.

### 3.3 Case 3

**Fig. 3:**
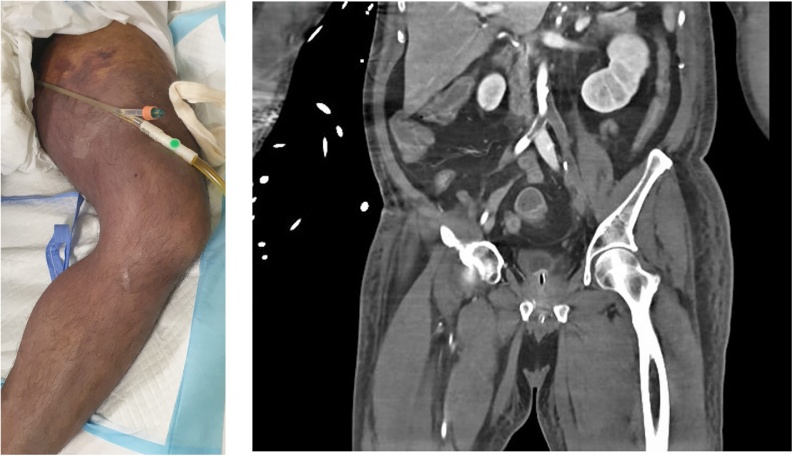
(a) Left lower limb with visible mottled and dusky appearance. (b) CT angiography, red arrow displaying significant occlusion aortic bifurcation and left common iliac artery.

A 60 year old hypertensive male presented to our institution with significant respiratory distress. He was subsequently intubated in the ER due to deteriorating respiratory status and reduced level of consciousness. On day 8 of his ICU course, the left lower limb appeared dusky and mottled up to the mid-thigh (Fig. 3). The right distal forefoot displayed a well demarcated discoloration.

CT angiography revealed; Infra-renal aortic left sided eccentric, non-calcified atheromatous plaque. Significant occlusion of the left common iliac artery extending to infra-popliteal arteries. A joint decision was made to avoid surgery given the poor prognosis associated with severe acute respiratory distress syndrome and high inotropic support. The patient expired the following morning.

### 3.4 Case 4

A 38 year old previously healthy male presented to our ER complaining of a 2 day history of worsening left arm pain and mild shortness of breath. On examination a discolored left arm was noted and markedly reduced/absent pulses. The patient was afebrile and the only abnormal laboratory investigations was a D-Dimer of 1500 ng/ml. CT-angiography showed a totally occluded left axillary artery and partial occlusion of the brachial artery - likely embolic. Bilateral pulmonary embolism noted on subsequent CT scan.

Heparin infusion was initiated and a surgical embolectomy performed successfully. The patient was admitted to the ICU for observation management. The patient recovered and was discharged after 2 weeks.

### 3.5 Case 5

**Fig. 4:**
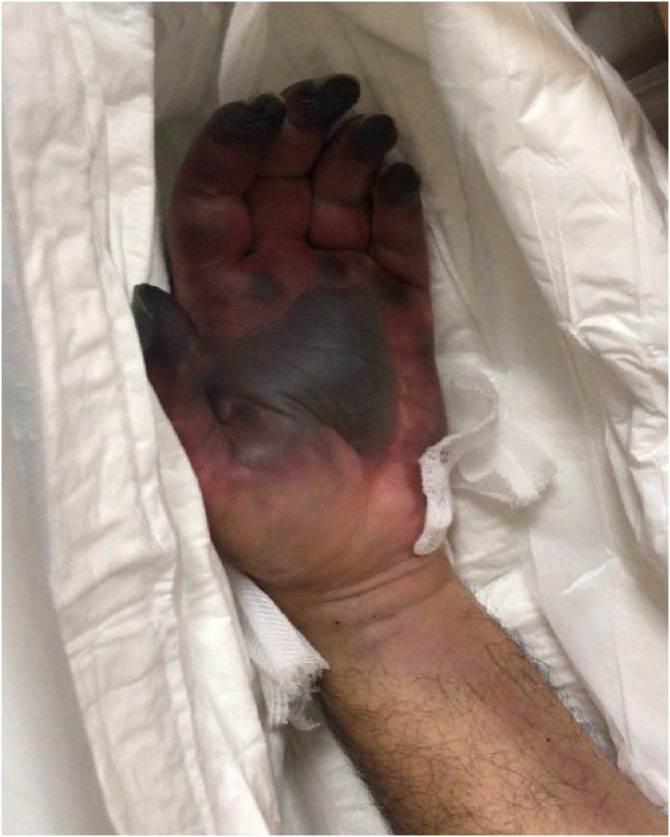
Left hand showing non-salvageable ischemia with significant gangrene.

A 58 year old previously healthy male who presented to us after being diagnosed with Covid-19 pneumonia. On arrival the patient was displaying signs of severe respiratory distress with exhaustion. He was immediately intubated and transferred to the ICU. During his stay he was on high ventilatory and inotropic support. On day 11, all distal extremities showed non salvageable ischemia with no palpable distal pulses (Fig. 4). Urgent CT angiography revealed extensive thrombosis affecting aortic bifurcation and bilateral iliac systems. Despite aggressive anticoagulation the patient subsequently expired secondary to refractory shock. The patient was anti-coagulated for the entire duration of his hospital stay (Table 1).

**Table 1:** Summarizing patient history, laboratory and radiological findings, management and outcome.

Variables	Patients				
	1	2	3	4	5
**Age**	38	48	60	38	58
**Gender**	Male	Male	Male	Male	Male
**Co-Morbidities**	None	Asthma	Hypertension	None	None
**Covid-19**	**Positive**				
**Lactate** Reference: 0.5–2.2 mmol/l	2.2	1.6	1.28	1.45	3.2
**D-Dimer** Reference: <500 ng//ml	3552	2100	2309	1500	1877
**CT**	PV, SV, SMA, IMA thrombosis.	Complete Left aorto-fem. artery occlusion	Infra-renal aortic left sided atheromatous plaque. left common iliac artery occlusion	Complete left axillary artery + partial brachial artery occlusion	thrombosis affecting aortic bifurcation and bilateral iliac systems
**Prophylactic** **Anticoagulation**	1^st^ presentation	Heparin Infusion	Heparin Infusion	1^st^ presentation	Heparin Infusion
**Vasopressors**	No	Noradrenaline	No	No	Noradrenaline
**Management**	Heparin Infusion + Resection & primary anastomosis	Heparin Infusion	Heparin Infusion	Heparin Infusion + surgical embolectomy	Heparin Infusion
**Outcome**	Discharged	Death	Death	Discharged	Death

*PV = Portal Vein, SV = Splenic Vein, SMA = Superior Mesentric Artery, IMA = Inferior Mesenteric Artery, INR = Internationaal normalized ratio, APTT = activated partial thromboplastin time.

## 4 Discussion

Our cases represent further evidence of the propensity for Covid-19 to present with unexpected vessel disease in seemingly healthy patients. These unusual cases should arouse clinical suspicion even in patients who are not predisposed to venous and arterial disease. The striking feature displayed is the thromboembolic propensity despite adequate prophylactic and therapeutic doses of anticoagulation.

In our institution; the main Covid-19 hospital in Kuwait. All of our patients received recommended doses of prophylactic and therapeutic enoxaparin, with some patients placed on heparin infusions upon admission based on existing recommendations and expert consensus. Our institution found that these cases differ significantly from patients suffering from distal extremity ischemia, confined to the distal phalanx and likely secondary to vasopressor infusions.

In Conclusion, despite the limited number of cases, we believe these cases to be significant additions and may aid further research. Presentations such as these are relatively rare. We believe Covid-19 plays a vital role in thromboembolic disease and warrants a high index of suspicion regardless of predisposing factors. A registry would need to be set up and further high level studies need to be carried out, to underline the etiology and treatment recommendations.

## Declaration of Competing Interest

The authors report no declarations of interest.

## Sources of funding

The research did not receive any funding.

## Ethical approval

The study is exempt from ethical approval in our institution Data observational study only.

## Consent

Consent from the patients and/or patient’s family was acquired where possible. Including consent to publish and include photography.

## Author contribution

Eisa Lari – Writing original draft, review and editing.

Ali Lari – Writing original draft, review and editing.

Shamlan AlQinai – Writing original draft, data collection.

Mahdi Abdulrasoul – Data collection.

Salman Al-Safran – Supervision, visualization.

Ahmed Ameer – conceptualization, reviewing.

Salman Al-Sabah – Conceptualization, Review.

## Registration of research studies

1. Name of the registry: www.researchregistry.com.
2. Unique identifying number or registration ID: 5853.
3. Hyperlink to your specific registration (must be publicly accessible and will be checked): https://www.researchregistry.com/browse-the-registry#home/registrationdetails/5f22d41692c55700169aa144/.

## Guarantor

Dr Eisa Lari.

## Provenance and peer review

Not commissioned, externally peer-reviewed.
